# Feasibility and efficacy of a novel technology‐based approach to harness social networks for weight loss: the NETworks pilot randomized controlled trial

**DOI:** 10.1002/osp4.352

**Published:** 2019-06-27

**Authors:** C. M. Monroe, M. Geraci, C. A. Larsen, D. S. West

**Affiliations:** ^1^ Arnold School of Public Health, Department of Health Promotion, Education, and Behavior University of South Carolina Columbia SC USA; ^2^ Arnold School of Public Health, Department of Epidemiology and Biostatistics University of South Carolina Columbia SC USA; ^3^ Arnold School of Public Health, Department of Exercise Science University of South Carolina Columbia SC USA

**Keywords:** eHealth, mHealth, social support, weight management

## Abstract

**Objective:**

Harnessing social support from existing social ties represents a key weight control practice. This trial evaluated an intervention that provided health‐promoting technologies for leveraging the influence of existing social ties.

**Methods:**

Volunteers (*N* = 36) with a body mass index between 25 and 55 kg m^−2^ were randomized to a 16‐week, in‐person, technology‐supported behavioural weight‐loss treatment (standard behavioural treatment) or the same programme supplemented by providing self‐selected members of participants' social networks with a digital body‐weight scale and Fitbit Zip physical activity tracker (ENHANCED).

**Results:**

Average weight losses from baseline to 16 weeks did not significantly differ between groups (standard behavioural treatment, 5.30%, SD =3.93%; ENHANCED, 5.96%, SD = 5.19%, *p* = 0.63). By the 1‐year follow‐up, standard behavioural treatment had lost 5.63%, SD = 8.14% of baseline weight versus 4.73%, SD = 9.43% for ENHANCED (*p* = 0.82). ENHANCED reported self‐weighing on more days than did standard behavioural treatment (*p* = 0.03). Most participants reported high programme satisfaction. Similar improvements were observed in perceived social support for diet and exercise from baseline to 16 weeks in both groups (*p*s < 0.05) but regressed by 1 year (*p*s < 0.01).

**Conclusion:**

Although feasible to implement, this technology‐based, social support approach failed to enhance outcomes of a face‐to‐face, group‐based behavioural weight‐loss treatment.

## 1 Introduction

Obesity is a persistent public health problem 1, 2. Exploring ways to enhance and expand the reach of existing best practices 3 for effective behavioural weight loss and maintenance is of paramount importance. Intensive multicomponent behavioural treatments targeting physical activity (PA) and diet represent the gold standard approach, yielding a clinically significant mean weight loss of 8–10% of initial body weight over 20–30 weeks 4. Yet higher long‐term weight loss is associated with an even greater benefit 5. Engaging social support is one key practice that has been shown to promote PA and dietary change, as well as weight control 3, 6, 7, 8, 9, 10, 11. However, methods to maximize social support for behavioural weight control have been explored only modestly.

Capitalizing on existing social ties represents one promising approach for enhancing social support for weight loss 6, 7, 8, 12, 13. For example, in one study, those participating in a social support‐enhanced behavioural weight‐loss intervention with friends achieved greater weight loss than did those in the standard behavioural treatment 6. Likewise, social support from pre‐existing social ties has been positively linked to PA 14 and eating behaviours 15.

Interventions targeting behavioural weight loss 16, 17, 18, 19, 20, healthy diet 21, 22 and PA 21, 22, 23, 24, 25, 26, 27, 28 have increasingly looked to online social media 16, 17, 22, 23, 24, 25, 26, commercially available dietary and PA apps 17, 18, 19, 20, 21, 22, 27, 28, and related wearable electronic PA trackers 18, 20, 21, 22, 27, 28 to enhance outcomes by promoting social support 16, 17, 19, 21, 22, 23, 24, 25, 26 and self‐monitoring of diet and PA 17, 18, 19, 20, 21, 22, 27, 28. This research has paralleled the growing popularity of commercially available PA‐focused and dietary‐focused smartphone apps and wearable PA tracking devices 29, 30. Over three quarters of U.S. adults report owning a smartphone 31, and recent data suggest that more than half of them have previously downloaded at least one health app, with PA tracking, dietary monitoring and weight‐loss apps representing the most popular types to be downloaded 29. Further, many of these apps work in concert with wearable PA monitoring devices to allow users to connect and interact with one another in real time across locations.

Although interventions that harness apps, wearables and social media have not always produced superior behavioural and weight‐loss outcomes than achieved with similar behavioural approaches that do not include this technology 16, 17, 18, 19, 20, 21, 22, 23, 24, 25, 26, 27, 28, few of the interventions tested to date integrated approaches designed to purposefully tap into the influence of existing social ties 23, 24, 25. Further, none have provided participants with health‐promoting interactive technology resources as an intentional way to engage and solicit support from their established social networks. Studies have shown that providing participants with tangible resources can improve weight‐loss and PA outcomes 13, 32. Further, few prior interventions have isolated the effect of the technology‐oriented social support elements 23, 26. Indeed, recent calls within the field of electronic and mobile health have been made to further (i) explore strategies that harness the joint influence of existing social ties and popular health‐related technologies 26, 33 and (ii) examine the isolated effect of specific intervention strategies to optimize behavioural interventions 34.

Thus, the purpose of this pilot study was to examine the feasibility and preliminary efficacy of a novel behavioural weight control approach designed to leverage the influence of existing social ties by providing health‐promoting technologies for increasing weight losses. It was hypothesized that engaging existing social networks via the provision of technologies would enhance treatment adherence, study retention and end‐of‐treatment weight loss among adults participating in a behavioural weight‐loss programme.

## 2 Materials and methods

### 2.1 Study design

A 16‐week, parallel‐group, pilot randomized controlled trial called NETworks (Nutrition, Exercise, and Technology works for weight loss) was conducted. Participants were recruited via flyers, targeted e‐mails sent via listservs and word of mouth for a technology‐supported weight‐loss programme between December 2015 and January 2016. The target sample size (*N* = 36) was primarily dictated by practical considerations, including budgetary constraints 35, and corresponds with observed median sample sizes of previous pilot and feasibility randomized controlled trials 36. Interested individuals applied through a study recruitment website and were screened by phone to identify likely eligible participants. These individuals were then invited to an in‐person orientation, where the study was described in detail, eligibility was confirmed and written informed consent was obtained. Baseline assessments occurred at a subsequent visit. Treatment assignment was revealed via email after the completion of all baseline assessments and prior to the first intervention session. Post‐treatment assessments were conducted at 16 weeks (end of intervention) and 1 year (follow‐up). Small incentives were offered for attending these assessment visits (i.e. socks and water bottles valued at $10 or less). The study was approved by the Institutional Review Board at the University of South Carolina.

### 2.2 Participants

Individuals were eligible if they were at least 18 years old, had a body mass index (BMI; kg m^−2^) between 25 and 55, were in general good health (no history of major disease), had access to the internet, owned a smartphone (iPhone or Android) and were willing and able to attend in‐person group counselling sessions on the predetermined designated day and time. Individuals were ineligible if they were pregnant or planning to become pregnant within 1 year of enrolment, currently breastfeeding, intending to move out of the Columbia, SC, area in the upcoming year, had diabetes or a medical contraindication for engaging in moderate‐intensity PA or weight loss, had a history of bariatric surgery, reported losing ≥10% of initial body weight within the previous 6 months, currently used medications that might affect weight loss or were enrolled in another weight‐loss programme. Only one member of a household was eligible to participate as an index participant.

### 2.3 Randomization

Participants were randomly allocated to either a 4‐month standard technology‐supported behavioural weight control treatment (SBT) or the same programme augmented by providing self‐selected members of each participant's social network with health‐promoting technology (ENHANCED), using a computer‐based random number generator in a 1:1 ratio. Although participants and interventionists were not blinded to treatment condition, outcome assessment was blinded.

### 2.4 Intervention

#### 2.4.1 Behavioural weight control programme

Both SBT and ENHANCED participants received the same evidence‐based group behavioural weight control programme focused on increasing PA and reducing calorie intake. This programme was patterned after successful weight‐loss programmes (Look AHEAD 37 and the Diabetes Prevention Program 38). It was delivered in 16 weekly, 60‐min, group counselling sessions and incorporated strategies derived from the social cognitive theory 39. Key behavioural modification strategies included self‐monitoring, problem solving, social support, goal setting, planning and relapse prevention. Group sessions were led by certified and experienced health promotion professionals (i.e. exercise physiologist and health education specialist). Participants were also asked to log in to a secure, password‐protected, responsive‐design study website to view individualized weekly feedback from the interventionist based on participants' self‐monitoring, a weight‐loss progress graph, weekly lesson information and homework assignments corresponding with topics addressed during the group counselling sessions, educational resources and group member profiles. All group counselling sessions took place in‐person except for one (week 15), which provided an interactive lesson on the study website due to a holiday conflict.

A calorie‐restricted diet based on initial body weight was prescribed, with calorie and fat gram goals ranging from 1,200 to 1,800 kcal d^−1^ and 33 to 50 g d^−1^, respectively. Graded PA goals that progressed to 10,000 steps d^−1^ and 200 min week^−1^ of moderate‐to‐vigorous intensity exercise were provided. All participants received a commercially available PA monitor (Fitbit Zip) and digital body‐weight scale (without wireless connectivity) to assist in self‐monitoring PA and weight, respectively. The Fitbit Zip is a small, waist‐worn activity tracker that continuously displays steps and distance travelled. It wirelessly syncs with a corresponding free app, allowing participants to track their PA over time, interact with other Fitbit users and engage with features designed to enhance social interaction around PA (e.g. badges, competitions and notifications). All participants were instructed to self‐monitor their dietary intake via the free version of a commercially available app (MyFitnessPal). This platform allows users to record calorie and fat goals and daily intake, track their progress towards their goals and interact with other MyFitnessPal users. During the first group counselling session, interventionists helped participants set up their Fitbit and MyFitnessPal accounts and provided guidance on how to use these technologies.

#### 2.4.2 ENHANCED intervention

In addition to this group‐based intervention, ENHANCED participants received two additional Fitbit Zips and two additional digital body‐weight scales to share with up to two adults (age ≥ 18 years) of their choice within their existing social network (i.e. support partners), with the intent of enriching their social climate for weight control behaviours. They were also provided with a brief invitation that they could use to formally invite these individuals to serve as their support partners. This invitation provided support partners with a description of the study, indicated that they would get to keep the provided Fitbit Zip and body‐weight scale, presented a short list of ways they could be supportive (e.g. become a Fitbit friend with the study participant; give encouragement; offer suggestions for recipes and places to exercise; and give compliments for goal achievements) and asked them to provide online consent to complete a survey about themselves. ENHANCED participants were strongly encouraged to take advantage of the Fitbit's embedded social support features (e.g. become Fitbit friends with support partners, cheer and send messages of support and engage in challenges if desired).

### 2.5 Outcome measures

#### 2.5.1 Treatment adherence

Interventionists recorded attendance at group sessions. Log‐ins to the study website were tracked during the 16‐week treatment. Total number of days that participants self‐monitored their diet and PA on MyFitnessPal and Fitbit, respectively, was calculated for both the 16‐week treatment period and the subsequent 36‐week follow‐up period. If participants logged two meals or snacks or more on a given day, self‐monitoring of diet was considered to have occurred that day. If any steps were registered on their Fitbit account for a given day, PA self‐monitoring was considered present for that day. Each week of the intervention, participants were also asked to report on the study website the number of days they weighed themselves. If they did not enter data for a week, they were considered to have not self‐weighed that week.

#### 2.5.2 Treatment satisfaction

At the end of the intervention, participants were asked via online questions about their satisfaction with the programme, likelihood of recommending it to others and its helpfulness in terms of facilitating an expanded social network for weight loss using a 5‐point Likert‐type scale. ENHANCED participants also rated the helpfulness of having support partners as part of their treatment.

#### 2.5.3 Body weight

Weight was measured to the nearest 0.1 kg in street clothes, without shoes, using a calibrated digital scale (Tanita BWB 800, Arlington Heights, IL) at baseline, weekly during intervention and at 1 year. Height was measured to the nearest 0.1 cm using a standard stadiometer at baseline only. BMI was calculated as weight (kg) per height (m^2^). The proportion who achieved clinically significant weight losses of ≥5% and ≥10% was also examined 40.

#### 2.5.4 Sociodemographic characteristics

Sociodemographic characteristics were reported at baseline via an online questionnaire.

#### 2.5.5 Social support for diet and exercise

Perceived social support from family and friends for diet (SSD) and exercise (SSE) were measured online using the valid and reliable Sallis Social Support Scales for Eating (10‐item scale) and Exercise (13‐item scale) Behavior 41 at baseline, 16 weeks and 1 year. Instructions were adapted to ask participants to rate each item on a 5‐point scale for perceived support from family and friends together. Item scores were averaged, with higher scores indicating a stronger sense of social support.

#### 2.5.6 Support partners

At baseline, support partners reported via an online questionnaire their sociodemographic characteristics, relationship status with the participant who invited them (friend or co‐worker; spouse, life partner or other relative), height, body weight and intentions regarding weight management (lose weight; gain weight; maintain weight; no intentions/not thinking about weight). BMI was calculated as weight (kg) per height (m^2^).

### 2.6 Statistical analyses

Descriptive statistics for baseline measures and retention rates were calculated for the sample, and descriptive statistics were also calculated for support partners (*N* = 33). Two‐sample *t*‐tests and two‐sample tests for proportions were used to analyse baseline and retention differences between conditions, as well as baseline differences between study completers and noncompleters. Primary outcomes were treatment adherence, study retention, treatment satisfaction and total weight change at 16 weeks. The changes in SSD and SSE from baseline to week 16 were secondary outcomes. Exploratory analyses were conducted to examine the proportion who achieved weight losses of 5% and 10% from baseline at week 16 and 1 year, as well as changes in weight, SSD and SSE at 1 year from baseline. Robust linear mixed effects models were used to examine changes in weight, SSD and SSE from baseline to 16 weeks (post‐treatment) and 16 weeks to 1 year (follow‐up). Maximum likelihood estimation for linear mixed effects models automatically handles missing responses under missing at random (expanded details on the analytic approach are available in Supporting Information). For all three outcomes (changes in weight, SSD and SSE), models were fitted using the R package robustlmm 42. With the use of multiple‐imputed datasets under missing at random, proportions of participants who lost at least 5% or 10% of their weight were compared in the two groups by means of log‐binomial regression. Missing weight data were imputed using multiple imputation by chained equations as provided by the R package mice 43. Each weight between week 1 and week 16 was imputed using two prior and two subsequent measurements, whenever available. Missing weights at follow‐up were imputed using weight at baseline and at week 16. Age, race, education, relationship status, height at baseline and BMI at baseline were used as auxiliary variables for imputation. Missing weights were imputed using linear models. The number of imputations and the number of iterations of the chains were both set to 10.

Two‐sample *t*‐tests were used to measure differences between groups for average number of group sessions attended, log‐ins, and self‐monitoring of PA, diet and body weight. Descriptive statistics were calculated for all satisfaction measures. Statistical significance was set at the 5% level. SPSS version 24.0.0 for Windows (IBM Corp., Armonk, NY) was used for analyses of treatment adherence and satisfaction data, as well as baseline measures and retention. R statistical software (R Core Team) 44 was used for all other analyses.

## 3 Results

**Figure 1:**
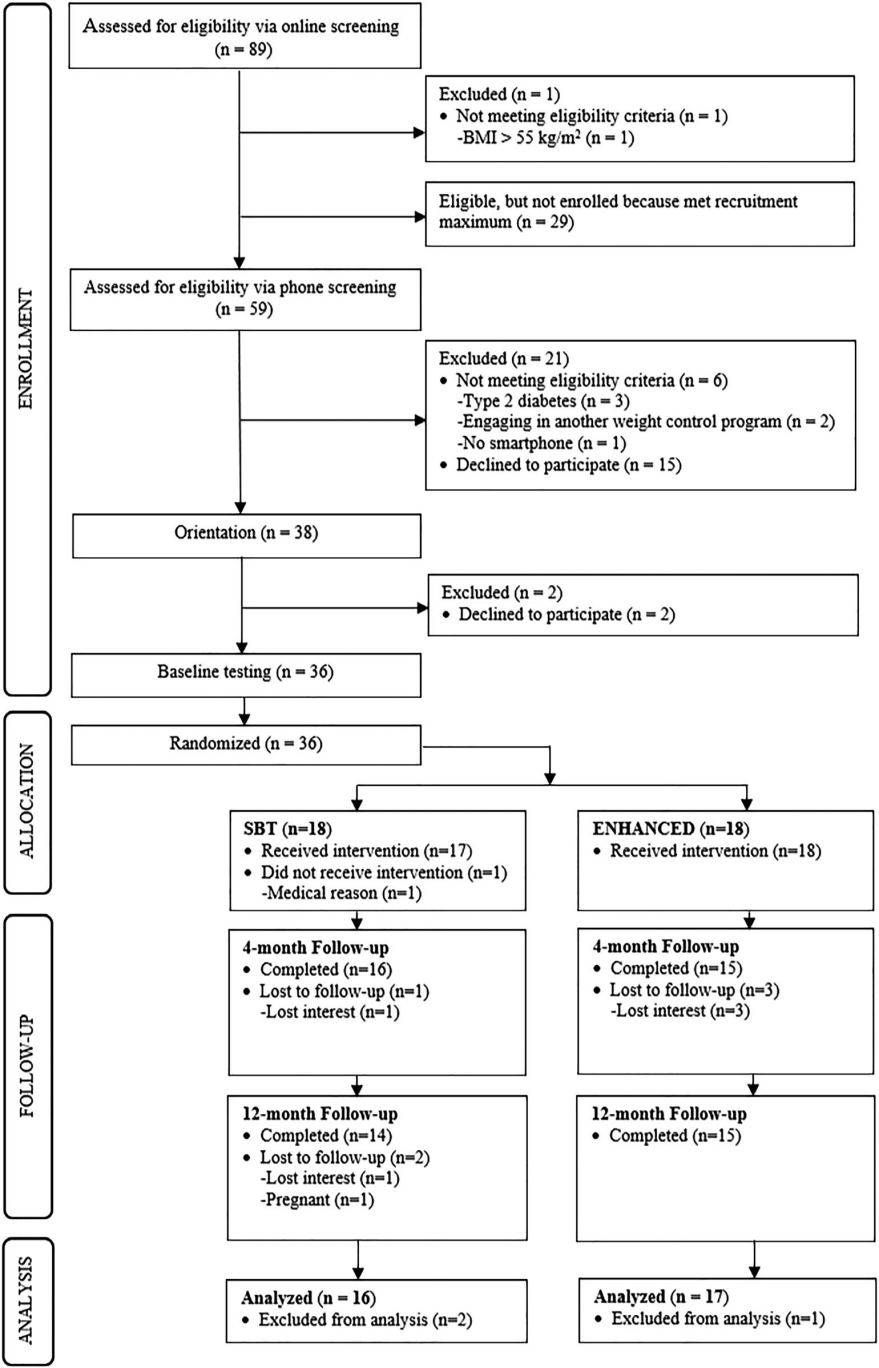
CONSORT flow diagram.

The flow of participants through the study is shown in Figure 1. A total of 36 participants were randomized to one of the two conditions. One SBT participant (female) did not begin the intervention owing to an injury unrelated to the study and was therefore removed from the analyses. One ENHANCED participant (the only man in the condition) was also removed from the analyses because he withdrew after the first introductory session. The single man randomized to SBT was removed from the analyses to eliminate a source of heterogeneity; therefore, the analytic sample consisted of women (*N* = 33). The pattern of results did not change by including all three of these participants in the analyses with the exception of adherence to the request to log in to the study website weekly during the intervention to report the number of days of self‐weighing, as well as the actual reported frequency of self‐weighing (data reported only for these self‐weighing‐related outcomes). Participants in the analytic sample did not differ at baseline on any parameter other than sex from those extracted from the full sample (*n* = 3; data not reported).

In addition to being composed only of women, participants in the analytic sample were mostly middle aged, White and well educated, with an average BMI in the obese range. There were no differences between conditions in baseline characteristics. Over 80% of participants were retained at 16 weeks and 1 year, with no difference between conditions in retention rates (Table 1). There were no significant differences in baseline variables between participants who provided follow‐up data and those who did not at either 16 weeks or 1 year (data not reported).

**Table 1:** Baseline characteristics and retention rates of analytic sample

Measure	All (n = 33)	Enhanced (n = 17)	Standard (n = 16)	p	Support partners (n = 33)^a^
Age, years	44.67 (8.96)	42.82 (9.09)	46.63 (8.68)	0.23	42.85 (12.96)^b^
Female, % (*f*)	100.00 (33)	100.00 (17)	100.00 (16)	1.00	77.42 (24)
Race, % (*f*)				0.13	
White	54.55 (18)	70.59 (12)	37.50 (6)		51.61 (16)
African–American	42.42 (14)	29.41 (5)	56.25 (9)		35.48 (11)
Asian	3.03 (1)	0.00 (0)	6.25 (1)		0.00 (0)
Mixed race	0.00 (0)	0.00 (0)	0.00 (0)		12.90 (4)
Education, % (*f*)				0.97	
Bachelor's degree or higher	93.94 (31)	94.12 (16)	93.75 (15)		80.65 (25)
Relationship Status, % (*f*)				0.62	
Married	54.55 (18)	47.06 (8)	62.50 (10)		48.39 (15)
Living as married	3.03 (1)	5.89 (1)	0.00 (0)		3.23 (1)
Divorced	15.15 (5)	17.65 (3)	12.50 (2)		12.90 (4)
Separated	3.03 (1)	0.00 (0)	6.25 (1)		0.00 (0)
Widowed	3.03 (1)	5.89 (1)	0.00 (0)		0.00 (0)
Single	21.21 (7)	23.53 (4)	18.75 (3)		35.48 (11)
Weight, kg	97.78 (21.04)	96.08 (24.36)	99.58 (17.45)	0.64	93.27 (29.04)^b^
BMI, kg m^−2^	36.22 (7.53)	35.61 (8.69)	36.86 (6.29)	0.64	33.74 (9.65)^c^
Social support for diet^d^^,^ e	3.14 (0.57)	3.31 (0.44)	2.97 (0.63)	0.09	
Social support for exercise^d^^,^ f	2.93 (0.80)	2.92 (0.67)	2.95 (0.93)	0.92	
Social support for diet^d^^,^ g	3.13 (0.57)	3.29 (0.46)	2.97 (0.63)	0.11	
Social support for exercise^d^^,^ g	2.95 (0.81)	2.92 (0.72)	2.99 (0.92)	0.85	
Retained for 16‐week follow‐up, %, (*f*)	90.91 (30)	88.24 (15)	93.75 (15)	0.58	
Retained for 1‐year follow‐up, %, (*f*)	84.85 (28)	88.24 (15)	81.25 (13)	0.58	

Data are mean (standard deviation) unless indicated by % (*f*).

a Data are based on *n* = 31 unless otherwise noted because 2 support partners chose not to complete the baseline survey.

*n* = 27.

b *n* = 30.

c *n* = 29.

d Score range 1 to 5, with 5 indicating high perceived support.

e *n* = 16 in ENHANCED group (1 responded does not apply).

f *n* = 13 in standard group (3 responded does not apply); *n* = 13 in ENHANCED group (4 responded does not apply).

g *n* = 33 (values imputed for missing data; used in analyses of social support).

Of the 18 participants randomized to ENHANCED, 16 selected two support partners, one selected a single support partner and one chose not to select any partners. Of the 33 support partners, 31 consented and completed the online questionnaire (93.94%). Support partners who provided information were mostly middle aged, well educated and female, with an average BMI in the class I obesity range (Table 1). Seventeen (54.84%) were friends or co‐workers, and the remainder were spouses or romantic partners (*n* = 2; 6.45%) or other relatives (*n* = 12; 38.71%). Twenty‐eight (93.55%) reported that they were currently trying to lose weight compared with two (6.45%) who were seeking to maintain weight and one (3.23%) who was not thinking about weight control.

### 3.1 Treatment adherence

The mean number of weekly group counselling sessions attended over the 16‐week treatment was 11.25 ± 3.61 for SBT and 13.06 ± 3.09 for ENHANCED, with no significant difference between conditions (*p* = 0.13). The mean number of log‐ins was also not significantly different between SBT and ENHANCED (23.00 ± 15.42 vs. 28.59 ± 15.90, respectively; *p* = 0.31).

Compared with SBT participants, those in ENHANCED had greater adherence to the request to log in to the study website weekly during the intervention (mean = 8.75 ± 5.03 vs. 12.47 ± 3.66 weeks out of 16 weeks; *p* = 0.02) to report the number of days they self‐weighed (mean = 46.31 ± 32.63 days vs. 72.52 ± 31.19 days out of 112 days for SBT and ENHANCED, respectively; *p* = 0.03). However, the pattern of these findings slightly changed when the full sample (*N* = 36; i.e. includes the three participants who were not part of the analytic sample) was considered in these analyses, with adherence to logging in to the study website weekly (mean = 8.61 ± 5.39 vs. 11.77 ± 4.60 weeks out of 16 weeks; *p* = 0.07) to report the number of days they self‐weighed (mean = 47.00 ± 35.61 days vs. 68.50 ± 34.75 days out of 112 days for SBT and ENHANCED, respectively; *p* = 0.08) becoming non‐significant.

The difference in the mean number of days of self‐monitoring dietary intake in MyFitnessPal was not statistically significant between groups during treatment (SBT, 78.06 ± 40.38; ENHANCED, 89.00 ± 25.87; out of 112 days; *p* = 0.37) or from the end of the programme to the 1‐year follow‐up (SBT, 60.50 ± 88.02; ENHANCED, 82.35 ± 99.19; out of 253 days; *p* = 0.51). Similarly, the average number of days of self‐monitoring PA did not differ between groups during treatment (SBT, 99.00 ± 19.66; ENHANCED, 99.00 ± 23.50; out of 112 days; *p* = 1.00) or afterwards (SBT, 136.06 ± 110.45; ENHANCED, 125.94 ± 107.04; out of 253 days; *p* = 0.79).

### 3.2 Treatment satisfaction

Both SBT and ENHANCED participants indicated a positive evaluation of their respective interventions, with 83.33% of SBT and 100.00% of ENHANCED participants indicating that they agreed or strongly agreed that they were satisfied with their programme and would recommend it to others. Both conditions also indicated that they felt the programme expanded their social network for weight loss, with 86.67% of ENHANCED participants and 91.67% of SBT participants responding that they agreed or strongly agreed with this statement. Most ENHANCED participants (80.00%) agreed that having support partners was helpful.

### 3.3 Weight change

Weekly weight losses during the 16 weeks of treatment averaged 0.31 kg week^−1^ in SBT (*p* < 0.001) and 0.36 kg week^−1^ in ENHANCED (*p* < 0.001). However, the difference between slopes (0.05) was not statistically significant (*p* = 0.63), indicating that the groups did not differ in their weekly weight loss over this period (Table 2). By post‐treatment (16 weeks), SBT participants lost an average of 4.93 ± 3.34 kg (5.30 ± 3.93%), and ENHANCED participants lost 5.63 ± 5.30 kg (5.96 ± 5.19%). The proportion of participants who had lost ≥5% at 16 weeks was similar between groups (42.42% for SBT vs. 46.36% for ENHANCED; *p* = 0.83). Although 21.44% of ENHANCED participants versus 8.25% of those in SBT lost ≥10% at 16 weeks, this difference was not statistically significant (*p* = 0.96) (Table 3).

**Table 2:** Linear mixed effects model for weight: estimates, standard errors and p‐values for the regression coefficients and estimated standard deviations of the random effects

Parameter	Estimate	Standard error	p‐value
*b*_0_	99.95	5.11	<0.001
*b*_1_	−6.64	7.12	0.36
*b*_2_	−0.31	0.07	<0.001
*b*_3_	0.29	0.10	<0.01
*b*_4_	−0.05	0.09	0.63
*b*_5_	0.08	0.13	0.55
*σ*_1_	19.93		
*σ*_2_	0.25		
*σ*_3_	0.34		
*σ*_*ε*_	0.73		

**Table 3:** Proportion of participants with weight loss ≥ 5% or 10%: comparison between standard (SBT) and ENHANCED groups in analysis with multiple imputation

Outcome	Multiple imputation			
	Estimate (proportions)		Estimate (log probability ratio)	p‐value
	SBT (n = 16)	ENHANCED (n = 17)		
≥5% (at 16 weeks)	42.42	46.36	0.09	0.83
≥10% (at 16 weeks)	8.25	21.44	0.96	0.39
≥5% (at 1 year)	44.68	45.14	0.01	0.98
≥10% (at 1 year)	27.84	32.72	0.16	0.78

Between the end of treatment and the 1‐year follow‐up, weight decreased on average by 0.02 kg week^−1^ in SBT (*p* = 0.85), suggesting that participants in SBT neither lost nor gained a significant amount of weight following the end of treatment. Over the same period, weight increased on average by 0.01 kg week^−1^ in the ENHANCED group (*p* = 0.90). However, the difference between slopes (0.04) was not statistically different between the two groups (*p* = 0.82), indicating comparable weight change over the follow‐up period (Table 2). The proportions of participants losing 5% and 10% at 1 year were comparable between the conditions, with no statistically significant differences (*p* = 0.98 and *p* = 0.78, respectively; Table 3). From study entry to the 1‐year assessment, SBT participants lost an average of 5.58 ± 8.23 kg (5.63 ± 8.14%), and ENHANCED participants lost 4.29 ± 8.84 kg (4.73 ± 9.43%).

### 3.4 Social support for diet and exercise change

Between baseline and 16 weeks, perceived SSD increased on average by 0.59 (SE = 0.21) in SBT (*p* = 0.01). In the same period, perceived SSD increased by 0.64 (SE = 0.20) in ENHANCED (*p* < 0.01). However, the difference between slopes (0.05) was not statistically significant (*p* = 0.88), suggesting comparable levels of social support for dietary change across the conditions. Between week 16 and 1 year, perceived SSD decreased on average by 0.58 (SE = 0.23) in SBT (*p* = 0.01). In the same period, perceived SSD changed by −0.72 (SE = 0.20) (i.e. decreased) in ENHANCED (*p* < 0.001). However, the difference between slopes (−0.14) was not statistically significant (*p* = 0.65) (Table 4; note that slopes and standard errors were adjusted to yield the total change from baseline to 16 weeks and the total change from 16 weeks to 1 year).

**Table 4:** Linear mixed effects model for social support for diet: estimates, standard errors and p‐values for the regression coefficients and estimated standard deviations of the random effects and error

Parameter	Estimate	Standard error	p‐value
*b*_0_	3.01	0.15	<0.001
*b*_1_	0.28	0.22	0.22
*b*_2_	0.04	0.01	0.01
*b*_3_	−0.05	0.02	<0.01
*b*_4_	<0.01	0.02	0.88
*b*_5_	−0.01	0.02	0.78
*σ*_1_	0.27		
*σ*_*ε*_	0.54		

Between baseline and 16 weeks, perceived SSE increased on average by 0.58 (SE = 0.27) in SBT (*p* = 0.04). In the same period, perceived SSE increased by 0.53 (SE = 0.23) in ENHANCED (*p* = 0.03). However, the difference between slopes (0.05) was not statistically significant (*p* = 0.91), indicating that changes in social support for exercise were similar for the two groups. Between 16 weeks and 1 year, perceived SSE decreased on average by 0.86 (SE = 0.28) in SBT (*p* < 0.01). In the same period, perceived SSE changed by −0.86 (SE = 0.22) (i.e. decreased) in ENHANCED (*p* < 0.001). There was no statistically significant difference between slopes (*p* = 0.96) (Table 5; note that slopes and standard errors were adjusted to yield the total change from baseline to 16 weeks and the total change from 16 weeks to 1 year).

**Table 5:** Linear mixed effects model for social support for exercise: estimates, standard errors and p‐values for the regression coefficients and estimated standard deviations of the random effects and error

Parameter	Estimate	Standard error	p‐value
*b*_0_	2.94	0.23	<0.001
*b*_1_	−0.03	0.33	0.94
*b*_2_	0.04	0.02	0.04
*b*_3_	−0.06	0.02	0.01
*b*_4_	0.00	0.02	0.91
*b*_5_	<0.01	0.03	0.92
*σ*_1_	0.58		
*σ*_*ε*_	0.59		

## 4 Discussion

The addition of a social support‐enhancement strategy (i.e. providing a Fitbit wearable PA tracker and a digital body‐weight scale to participant‐selected support partners) to a standard group‐based behavioural weight‐loss treatment did not increase weight losses in the short or long term over and above what was achieved with the standard treatment alone. Indeed, the simple provision of this ‘connected’ technology to members of an individual's social network within the context of a standard lifestyle programme failed to drive greater improvements in retention, self‐monitoring of diet and PA, programme attendance or perceived social support for diet and exercise from friends and family than seen with the standard programme alone. Nevertheless, the enhanced programme proved to be feasible and acceptable, with all but one participant sharing a study‐provided Fitbit and scale with self‐selected support partners, most indicating that incorporating support partners was helpful and all expressing satisfaction with their overall treatment.

The behavioural weight control programme produced clinically significant weight losses (≥5%) 40 in a substantial proportion of the participants in both groups and is comparable with what has been observed in other studies implementing a similar lifestyle intervention. In contrast, the weight‐loss trajectories of participants over the follow‐up period after 8 months without treatment did not follow the pattern commonly observed in other studies. In most programmes without a formal weight maintenance component, weight regain occurs once intervention sessions cease 4. However, in the current study, weight regain was not apparent; indeed, a higher proportion of participants in both conditions had achieved a 10% weight loss at 1 year than was observed at 16 weeks. This pattern is unusual and was manifest in both conditions. The present intervention is the first report to date to provide weight‐loss outcome data from healthy individuals who are provided a Fitbit as part of a structured, in‐person behavioural weight control intervention and given instructions on how to incorporate the wearable PA tracker in their weight‐loss efforts. Self‐monitoring is consistently associated with better weight losses 45. Self‐monitoring of PA post‐treatment remained high among participants in both study arms, even though the intervention had stopped and no counsellor feedback was being provided, with participants monitoring on over half the days during this period; nevertheless, it is unclear whether the Fitbit or other factors facilitated this continued level of robust self‐monitoring in the absence of treatment. The effectiveness of such devices for influencing sustained behavioural and health improvements requires further exploration.

The ineffectiveness of the enhanced social support strategy for improving weight losses, most treatment adherence indicators, retention and social support beyond best practices contrasts with previous studies that have leveraged existing social ties with success 6, 13. For example, Wing and Jeffery 6 observed greater weight losses and improvements in social support among those who participated in a behavioural weight‐loss programme with friends, co‐workers or family members than among those who participated alone. In another study 13, participants who engaged in a behavioural weight‐loss treatment with a support partner and also received tangible resources to assist with their weight management efforts (e.g. cookbook, food scale, treadmill or stationary bicycle and digital body‐weight scale) achieved greater weight losses at 6 months relative to those who participated alone and did not receive resources (9 vs. 7 kg, respectively). The current study provided participants in the standard behavioural intervention with the interactive technologies, although the provision of these resources was limited to the index participant. However, it may well have been that participants in the standard group received support from their existing social ties who already owned a Fitbit or who used the freely available apps, thus contaminating the standard group with respect to the social support manipulation. This potential factor might account for the comparable changes in perceived social support, which were observed across the conditions and suggests the need for careful consideration of how technology is integrated into the comparison condition in future research when trying to isolate the impact of enhancing social networks.

Although most support partners in the present study reported being overweight and trying to lose weight, they were not targeted for treatment, nor was there specific outreach to them other than a brief introduction that was focused on the index participant rather than on their own behaviour change; this fact may help explain differing outcomes in relation to these previous studies in which the support partners were integrated into the treatment 6, 13. Support partners in the current study may have felt detached from the programme and, thus, been less inclined to provide effective support and less likely to experience successful weight loss themselves 46. Weight change among the partners was not assessed in the current study. However, other researchers have noted the high correspondence in weight loss between partners and participants, suggesting that support partners losing weight promotes weight loss in participants (and vice versa) 7, 46, 47, 48. It is also possible that support partners did not know how best to support participants as they were only given general guidance and no additional training or feedback during the course of the programme, thus diminishing the potential impact of their role as a support partner 49. Future studies seeking to potentiate existing social ties should aim to capture and characterize social processes, behaviours and weight trajectories from both the participant's and support partner's perspectives. Moving forward, formal support partner training that addresses supportive communication styles, caters to the support needs of the participants, provides guidance on delivering support through technologies and helps the partners feel more involved may represent an effective addition to the social support‐enhancement strategy employed in the present study 49, 50.

Participants in the enhanced intervention were more engaged with the suggestion to weigh themselves daily, reporting that they weighed themselves on more days than those in the standard condition. Notably, this finding became non‐significant when considering the full sample (i.e. *N* = 36; includes the three participants who were not part of the analytic sample), although the absolute difference between groups in this reported outcome remained similar. Although daily self‐weighing has been demonstrated to improve weight losses in other studies 45 and appears to have a particular role in facilitating weight maintenance 51, the level of self‐weighing resulting from the enhanced intervention did not translate to better weight‐loss outcomes. Participants and partners treated together in a previous study identified the body‐weight scales provided as very helpful for their weight loss 13. The current study did not directly query about the role of the provided scales, nor did we examine how the participant–partners teams interacted around self‐monitoring their weight. Future studies should assess underlying social mechanisms that may be driving utilization (or lack of utilization) of technology or other resources when provided to social networks.

Limitations of this study include a small analysed sample composed only of highly educated women, reducing the generalizability of the findings, and the potential contamination across conditions if those in the standard group had members of their social circle using Fitbits that were not provided by the study. Given that approximately 40 million US adults reported owning a wearable PA monitor in 2016, and Fitbit devices accounted for almost two‐thirds of these 52, this is quite likely. Further, data characterizing support partners were collected at baseline only, limiting insights into the processes of social support across the duration of the yearlong study, and weight change in the partners was not characterized. Finally, data on the frequency of self‐weighing were self‐reported. However, PA and dietary self‐monitoring rates were objectively measured.

Augmenting a face‐to‐face, group‐based behavioural weight‐loss treatment with an approach designed to harness social support inherent within existing social ties by providing interactive technological resources to the extended social network was feasible but failed to enhance weight losses. The continued proliferation of interactive health‐promoting PA trackers and their associated smartphone apps, as well as the demonstrated influence of existing social ties on health, warrants continued evaluation of methods that exploit the intersection of technology and social support.

## Funding

This study was supported by an ASPIRE grant from the Office of the Vice President for Research‐University of South Carolina and funds from the Technology Center to Promote Healthy Lifestyles‐University of South Carolina.

## Conflict of Interest Statement

The authors have no competing interests.

## Supporting information


**Data S1.** Supporting information
