# Supplementary material for: Feasibility and efficacy of a novel technology‐based approach to harness social networks for weight loss: the NETworks pilot randomized controlled trial
Source: Obes Sci Pract. 2019 Jun 27;5(4):354–65. doi: 10.1002/osp4.352 (PMC6700517; doi:10.1002/osp4.352)
Supplement: Supplementary file 1 — Data S1. Supporting information [file OSP4-5-354-s001.pdf]

Feasibility and efficacy of a novel technology-based approach to harness social networks for weight loss: The NETworks pilot randomized controlled trial

Courtney M. Monroe, PhD
University of South Carolina
Arnold School of Public Health
Department of Health Promotion, Education, and Behavior
915 Greene Street, Discovery 1 Building, Suite 403G
Columbia, SC 29208
cmmonroe@mailbox.sc.edu

Marco Geraci, PhD
University of South Carolina
Arnold School of Public Health
Department of Epidemiology and Biostatistics
915 Greene Street, Discovery 1 Building, Suite 435C
Columbia, SC 29208
geraci@mailbox.sc.edu

Chelsea A. Larsen, MPH
University of South Carolina
Arnold School of Public Health
Department of Exercise Science
915 Greene Street, Discovery 1 Building, Suite 403
Columbia, SC 29208
calarsen@email.sc.edu

Delia Smith West, PhD
University of South Carolina
Arnold School of Public Health
Department of Exercise Science
915 Greene Street, Discovery 1 Building, Suite 403C
Columbia, SC 29208
westds@mailbox.sc.edu

Expanded details on the statistical analyses of changes in body weight and perceived social support for diet (SSD) and exercise (SSE)

To test the null hypothesis of no difference between groups in temporal trajectories of weight, we fitted the following linear mixed effects (LME) model:

$$weight = b_0 + \ b_1 \cdot trt + b_2 \cdot time + b_3 \cdot time16 + b_4 \cdot trt{:}time + b_5 \cdot trt{:}time16 + u_1 + time \cdot u_2 + time16 \cdot u_3 + \varepsilon \qquad (1)$$

where *time* is the variable time between baseline and 16 weeks, and *time*16 is the variable time between 16 weeks and 1 year. This model allows for two different slopes between baseline and 16 weeks and between 16 weeks and one year. The coefficients b_2 and ($b_2 + b_3$) measure the change in weight (baseline to 16 weeks and 16 weeks to 1 year, respectively) during those two time intervals in SBT, while ($b_2 + b_4$) and ($b_2 + b_3 + b_4 + b_5$) measure the change in weight in ENHANCED (trt). The random intercept u_1 and random slopes u_2 and u_3 give individual deviations from the overall temporal trajectory. Due to the relatively small sample size, we used a diagonal matrix to model the variance-covariance of the random effects. Analogously, two LME models of the type

$$SS = b_0 + \ b_1 \cdot trt + b_2 \cdot time + b_3 \cdot time16 + b_4 \cdot trt{:}time + b_5 \cdot trt{:}time16 + u_1 + \varepsilon \qquad (2)$$

were fitted to model temporal trajectories in SSD and SSE. Only a random intercept was included in the models given the estimates of the variances of the random slopes were approximately zero for both SSD and SSE.
